# Suprascapular nerve injury during robot-assisted radical prostatectomy: a case report

**DOI:** 10.1308/rcsann.2022.0148

**Published:** 2023-02-07

**Authors:** L Drummond, A McNeill

**Affiliations:** Western General Hospital, UK

**Keywords:** Suprascapular Nerve, Injury, Robot, Body Mass Index

## Abstract

This case report aims to encourage teams to readjust the operating table during prolonged robotic cases, especially in those patients with a high body mass index. We present the case of a 59-year-old male who developed neuropathic pain involving the suprascapular nerve distribution following a prolonged robotic-assisted radical prostatectomy.

## Case history

This case discusses a 59-year-old male with localised Gleason 3+4 prostate cancer, PSA 21, T2N0M0. Height 170cm, weight 103.8kg, body mass index (BMI) 35.92.

This patient chose robot-assisted radical prostatectomy after counselling regarding all treatment modalities.

Robotic-assisted radical prostatectomies are carried out in our unit using the Da Vinci Si robot. Patients are positioned supine and head down; head and neck in the neutral position; both arms by their sides. A specially designed cushioned head and shoulder support (Trendelenburg Head Cushion) is placed to prevent the patient slipping down the table. Routinely this operation takes 2–3h during which time any pressure on the shoulders is usually diminished by this cushion.

This patient had a history of neck pain for which he had two previous magnetic resonance imaging (MRI) scans: one in 2016, revealing significant degenerative changes throughout the cervical spine; multilevel cervical disk/osteophyte bulging causing bilateral foraminal narrowing at C3/4 and C4/5 but no cervical canal stenosis or cord compression; and one in 2020, revealing no further degeneration.

His prostatectomy was completed on an elective list, with a team well versed in the set-up and functionality of the robot; it was uncomplicated but difficult due to his body habitus, and as a result took 4h.

Immediately following the operation, he reported significant right arm pain with mild left arm pain.

On right upper limb examination he had 3/5 power over myotomes C6/7/8/T1, with normal tone but pain on passive movement of myotomes C6 and C7. He had forearm paraesthesia conforming neither to a nerve route nor peripheral nerve distribution, and normal coordination. Left arm examination revealed 4/5 power over myotomes C6/7/8/T1, otherwise identical examination to the right. Pain failed to improve with analgesia, concerning for compartment syndrome.

On review by the orthopaedic team, all compartments were soft with pressure points over both trapezius regions and pain on all movements of the neck. Pain on passive movement resolved while power improved to 4/5 on right and 5/5 on left. Paraesthesia now localised to C7/8/T1 on the right and had resolved on the left.

His right arm pain worsened 15h postoperatively, requiring increasing doses of gabapentin, oxycodone and a ketamine infusion. Power in the arm was maintained at 4/5.

Over the following 72h his pain improved; the ketamine infusion was stopped.

He was then reviewed by the neurosurgical team, at which point pain was triggered only on active movement and paraesthesia was resolved entirely. Power on the right improved, with 4/5 power over T1, otherwise 5/5 elsewhere.

MRI cervical spine and brachial plexus was completed, showing no progression of the known degenerative changes. There was no brachial plexus impingement. In keeping with denervation changes, oedema was present in the supraspinatus and infraspinatus muscles bilaterally, with no atrophy. Suggestion of bilateral suprascapular nerve involvement without evidence of compressive lesion on either side.

The impression was of muscular trauma causing neuropraxia; this was due to compression caused by prolonged head-down positioning, confounded by the high BMI of the patient.

One week postoperatively, he was discharged with regular oxycodone and gabapentin. Nerve conduction studies are awaited.

## Discussion

The suprascapular nerve is formed from the C5 and C6 nerve roots, ie, the upper trunk of the brachial plexus. Its route passes deep to the trapezius muscle, traverses laterally along the superior border of the scapula, providing two branches to the supraspinatus muscle before passing beneath the superior transverse scapular ligament and through the suprascapular notch into the supraspinous fossa. Here, it provides two terminal branches to the infraspinatus muscle. Supraspinatus and infraspinatus are both members of the rotator cuff muscles, the others being teres minor and subscapularis, and all being involved in stabilising the glenohumeral joint. The supraspinatus muscle, which lies deep to trapezius, functions in conjunction with the deltoid muscle to abduct the humerus at the glenohumeral joint. The infraspinatus muscle is responsible for external rotation of the upper limb at the glenohumeral joint.^1-3^

Intraoperative nerve injuries can occur due to a number of different mechanisms: compression causing interruption in blood flow to the nerve or increased intraneural pressures and resultant oedema; stretch or shearing force causing injury to the intraneural connective tissue; or complete disruption of the nerve. The extent of this injury depends on the duration of the interruption to blood supply and the severity of compression, and degree of resultant oedema. Sustained insult leads to myelin and Schwann cell damage, and subsequent recovery via remyelination can take several weeks, whereas a brief interruption in blood supply may be reversed within a matter of minutes on reperfusion.^4^ In 2013 Mills *et al* published a retrospective review of 334 patients who had undergone a robot-assisted urological procedure. They documented 22 position-related nerve injuries and found that 59.1% (*n*=13) of these neuropathies had resolved within 1 month while 22.7% (*n*=5) had not resolved at 6 months postoperatively. The main risk factor cited was operation duration.^5^ Indeed, Warner *et al* in 1994 proved, with respect to lower limb neuropathies, that a patient’s risk increases 100 fold with each hour they spend in the lithotomy position.^6^ In 2012, Koç *et al* confirmed these findings when they published a retrospective review of 377 patients who had undergone a robot-assisted laparoscopic prostatectomy in which they focused on postoperative lower extremity neuropathies. They reported the only variable identified as a significant and consistent risk factor was increased operative time. They examined multiple further variables including, height, weight, BMI, age, smoking status and surgeon’s experience; however, found none of these factors to increase the patient’s risk with respect to lower limb neuropathy.^7^ However, this does not appear to be the case with upper limb neuropathies. A high BMI (≥25kg/m^2^) has been shown to be a risk factor, increasing the compression pressure when the patient is in the head-down Trendelenburg position.^8,9^ This pressure can be reduced through appropriate use of gel pads,^10^ such as the specially designed Trendelenburg Head Cushion. Nerves in patients with peripheral vascular disease have a reduced baseline perfusion and thus require a lower degree of insult to cause an injury, making vascular disease another important patient-based risk factor. Diabetes and smoking are also risk factors, due similarly to reduced perfusion and thus diminished baseline quality of peripheral nerves.^6,8^ Indeed, Warner *et al* report smoking as increasing a patient’s risk by 5-fold.^6^ Of these identified risk factors, the most readily modified is the length of time spent in the head-down position. Although operative time is not directly controllable, the duration of the position can be. Checkpoints could be set at agreed timings; every 2h, consideration could be given to temporary table-levelling and assessment of pressure points. Indeed, continuous intraoperative neuromonitoring was described by Watson *et al* to monitor for potential nerve injury and invoke positional changes.^9^ Patient factors could also be modified, to an extent, through judicious diabetic control, weight loss management and smoking cessation advice.

## Learning points

Identify the risk: it is important to be aware of the risks of the prolonged head down position during operations and to identify additional patient factors that compound this risk, including high BMI, diabetes and smoking. A natural pause could be planned between the surgical and anaesthetic team to relax the head-down position midprocedure in an attempt to reduce patient risk. Equipment technology has moved on and the integrated table motion system available with the newer Da Vinci XI system will allow for the head-down position to be relieved more easily at intervals (2h) without undocking all of the instruments. Furthermore, this case report brings to light the importance of informing patients of the risk of peripheral nerve injury due to operative positioning.
